# Topical application of *Cassia fistula* L. fruit gel in management of cutaneous lesions of pemphigus vulgaris: A double-blind, placebo-controlled clinical trial

**Published:** 2018

**Authors:** Fatemeh Atarzadeh, Mohammad Kamalinejad, Gholamreza Amin, Alireza Salehi, Ladan Dastgheib, Amir Mohammad Jaladat, Mojtaba Heydari, Zahra gouyandeh

**Affiliations:** 1 *Department of Traditional Iranian Medicine, School of Traditional Medicine, Tehran University of Medical Sciences, Tehran, Iran*; 2 *School of Pharmacy, Shaheed Beheshti University of Medical Scinces, Tehran, Iran*; 3 *Department of Pharmacognosy and Department of Traditional Pharmacy, Tehran University of Medical Sciences, Tehran, Iran*; 4 *Research Center for Traditional Medicine and History of Medicine, Shiraz University of Medical Sciences, Shiraz, Iran*; 5 *Shiraz Molecular Dermatology Research Center, Department of Dermatology, Shiraz University of Medical Sciences, Shiraz, Iran*; 6 *Department of Traditional Persian Medicine, School of Medicine, Shiraz University of Medical Sciences, Shiraz, Iran*

**Keywords:** Cassia fistula L., Pemphigus vulgaris, Traditional Persian Medicine, Topical therapy

## Abstract

**Objective:**

*Cassia fistula* L. fruit extract has been traditionally used in the treatment of pemphigus vulgaris (PV) lesions in Iran. The aim of this study was to determine the efficacy of *C. fistula* fruit gel on healing time of PV lesions in a clinical setting.

**Materials and Methods:**

This was a randomized, double-blind placebo-controlled clinical trial that was performed in dermatology ward at Saadi hospital, affiliated to Shiraz University of Medical Sciences, Shiraz, Iran. Right- or left- sided lesions of PV patients on standard systemic treatment were randomized for treatment with either *C. fistula* fruit gel or placebo prescribed twice daily. The largest diameter of each lesion was measured at the baseline (day 0) and on days 10 and 20. Epithelialization Index (EI), as outcome measure was calculated and compared between the two groups.

**Results:**

The present study comprised 20 patients, with overall 82 cutaneous lesions including 41 lesions in the *C. fistula* fruit gel group and 41 lesions in the placebo group. The EI in the *C. fistula* fruit gel group was significantly higher than that of the placebo group both on day 10 (65±28vs 30±34; p=0.001) and at the end of the study (91±22 vs 69±49; p=0.003).

**Conclusion:**

Topical application of *C. fistula* fruit gel can be considered as an effective adjuvant therapy in treatment of PV.

## Introduction

Pemphigus vulgaris (PV) is a rare blistering autoimmune disease of the skin and mucus membranes with poor prognosis. It could be caused by immunoglobulin G (IgG) autoantibodies that bind desmoglein 1 and 3 surface antigens of the keratinocytes which are a structural part of desmosome cell to cell adhesion, resulting in acantholysis and skin and mucosal blister formation (Kershenovich et al., 2014 ▶; Bystryn and Rudolph, 2005 ▶). It appears that oxidative stress is a main player of the onset and exacerbation of PV. There are striking associations between the amount of serum oxidative stress markers and serum levels of anti-desmoglein antibody in pemphigus (Abida et al., 2012 ▶). Other factors involved in the pathogenesis of PV are the leukotrienes (LTs), since LTB4 and LTC4 levels in the skin are highly elevated under different inflammatory cutaneous disorders, including PV (Sadik et al., 2013 ▶).

 The typical findings of PV are flaccid blisters (Bruckner-Tuderman and Stanley, 2008 ▶) which are easily torn and painful pruritic erosions are formed. The healing process is slow (Ruocco et al., 2013 ▶) and systemic corticosteroids are the main treatment of PV. The use of corticosteroids and immunosuppressive drugs has reduced the mortality rate to less than 15% (Iraji and Banan, 2010), but serious morbidities related to side-effects of drugs, are still common (Anhalt and Díaz, 2004 ▶). 

 Patients with PV are susceptible to skin infections that could lead to severe sepsis and death. Thus, local skin care plays an important role in management of patients with PV and helps early epithelialization and alleviates pain and discomfort (Tabrizi et al., 2007 ▶).

It has been shown that shortening the healing time of erosions can reduce the period of active phase of the disease, risk of infection and duration of hospitalization (Calebotta et al., 1999 ▶).

Many studies on PV have, therefore, focused on the use of topical non-steroid agents as pimecrolimus (Iraji et al., 2010), nicotinamide (Iraji and Banan, 2010 ▶) and epidermal growth factor (Tabrizi et al., 2007 ▶).

The cost of these drugs and the risk of systemic absorption necessitate further trials to find new low cost, safe and effective topical treatment adjuvants to accelerate the healing process of pemphigus erosions (Skowron et al., 2005 ▶).

*Cassia fistula *L. has been traditionally used in the treatment of HOT nature skin lesions (inflammatory skin lesions such as burning, cutaneous leishmaniasis and pemphigus lesions) in Traditional Persian Medicine (Aghili Khorasani, 1388 ▶; Jaffary et al., 2006 ▶; Jaffary et al., 2010 ▶; Atarzadeh et al., 2016b ▶, 2016c ▶; Atarzadeh et al., 2017a ▶). It has long been used both orally and topically for healing of wounds and burns in the folk medicine by the tribal communities of various countries (Kumar et al., 2006 ▶; Ayyanar and Ignacimuthu, 2009 ▶).

*C. fistula* fruit gel can be used as a herbal medicine for treatment of PV erosions, because of its anti-oxidant (Rajagopal et al., 2013 ▶), anti-inflammatory (Anwikar and Bhitre, 2010 ▶), and anti- leukotrienes' effects via inhibition of 5- lipoxygenase mediated peroxidation of arachidonic acid (Rizvi et al., 2009 ▶), moreover, it can block secondary wound infections due to its antibacterial and antifungal activity (Bhalodia et al., 2012 ▶).

As far as we know, no clinical trials have been conducted on the wound healing efficacy of *C. fistula *L. against PV lesions. The purpose of this randomized, double-blind study was to evaluate the efficacy of topical *C. fistula* fruit gel in accelerating the healing process of skin lesions in patients with PV. 

## Materials and Methods

**Trial design**

This was a randomized, double-blind placebo-controlled clinical trial that was performed in dermatology ward at Saadi hospital, a referral hospital affiliated to Shiraz University of Medical Sciences, Shiraz, Iran from September 2013 till December 2014.

As no previous study had evaluated the potential of *C. fistula* for treating pemphigus, sample size was determined on the bases of similar topical works on pemphigus wound healing and the number of patients admitted to Saadi Hospital during our project. 

**Eligibility criteria**

Twenty patients with PV diagnosed by both histological and direct immunofluoroscent (DIF) evaluations, were enrolled into this study. Pregnant women, nursing mothers and those with a known history of any adverse reaction to the materials used, patients with infected skin lesions and facial skin lesions were excluded from this study. Only patients who had at least two symmetrical lesions on the trunk or upper or lower extremities, were included in the study. A total of 82 erosions (41 in each group) located on the trunk or upper or lower limbs were included.

**Ethical considerations**

The study was ratified by the Ethics committee of Research Institute for Islamic and Complementary Medicine (approval No. 114/TM/26/P). Furthermore, the trial was registered in the Iranian Registry of Clinical Trials (IRCT2013120815720N1). Before starting the study and after providing the patients with necessary information, informed consent was signed by each participant.

**Preparation of** ***Cassia fistula*** **L.**

*C. fistula *L. dried fruit was purchased from local market in Tehran bazaar, the center of Tehran province, Iran, identified by Professor Gholamreza Amin, and kept at the herbarium (voucher No. PMP- 653) of faculty of pharmacy, Tehran University of Medical Sciences.

**Preparation of gel and placebo**

Here, 25 grams of fruit pulp of *C. fistula* was boiled with 200 ml sesame oil and 200 ml water until the whole water was evaporated; then, it was cooled and filtered to be mixed with 934 carbapol (PubChem CID: 6581). The medication was prepared in gel form based on the 8.3% concentrated pulp of *C. fistula* equivalent to 33% condensed oil [obtained from *C. fistula*]. Placebo was prepared from 934 carbapol polymer with natural yellowish color purchased from Hakim Pharmaceutical Company. The final products were packed in similar tubes with the specified labels.

**Interventions**

All patients received standard treatment which mainly comprised of systemic prednisolone 1 mg/kg and an adjuvant as azathioprine or mycophenolate mofetil, which were continued throughout the trial. 

 Skin lesions on one side were randomly regarded as the control (i.e. the placebo group), and on the other side, as the intervention group (treated with *C. fistula* fruit gel). The drug and the placebo had the same color, texture and smell and were packaged in comparable tubes with different labels of A and B. Both patients and investigators were blinded to the end of the study. Half body design (each patient being his/her own control) was performed to remove the confounding effects of different systemic therapy regimen given to each patient.

**Outcome**

The lesions were examined on days 0, 10 and 20. In each examination, the largest diameter of the lesions was measured by a caliper and ruler and any adverse effects were recorded. To evaluate the healing effect of the drug and placebo, the Epithelialization Index (EI) was calculated for each skin lesion on days 10 and 20 after initiation of therapy using the following formula:

$\mathrm{EI}=.\frac{\begin{array}{c} (\mathrm{Maximum diameter at the baseline}–\\ \mathrm{Maximum diameter at the end of study}) \end{array}}{\mathrm{Maximum diameter at the baseline}}\times 100$

**Blinding**

A computer-generated randomization list was made by one of the authors who selected one or more appropriate pairs of skin lesions on each side of the patients’ bodies. This means that the application of the medication or the placebo was done based on randomization found within the list handed over to the ward nurse. The diameter of each erosion was determined initially and after potassium permanganate bath, a thin layer of drug or placebo was applied on the erosion twice daily for 20 days by a trained nurse. 

The code of the drugs was revealed at the end of the study and the results were analyzed.

**Figure 1:**
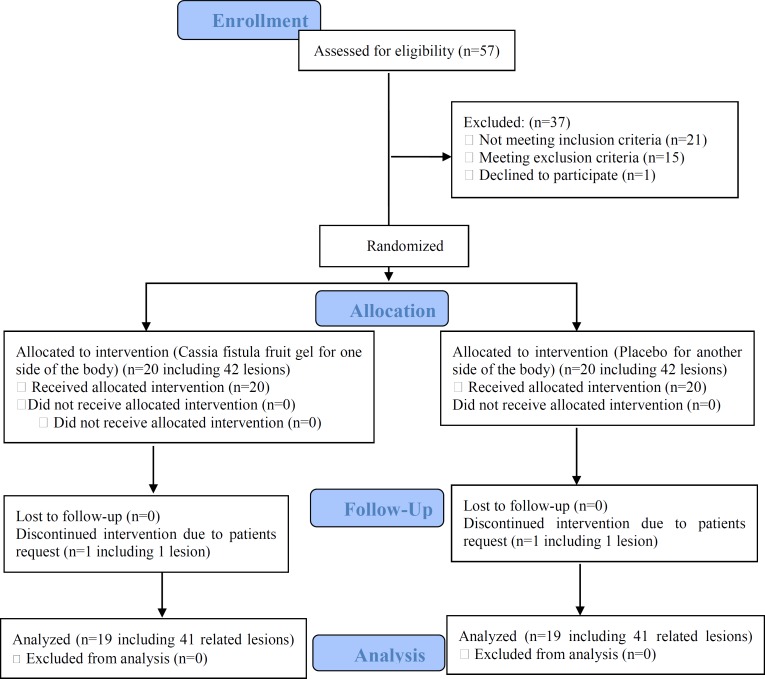
Study flow chart showing randomization and treatment assignment

**Statistical analysis**

The descriptive data were presented as means±standard deviations for quantitative and percentage for qualitative data. Friedman and Wilcoxon tests were used for statistical comparison of primary characteristics and outcomes in each group and between the drug and placebo groups. A p-value of less than 0.05 was considered statistically significant.

## Results

Figure 1 shows the study flow diagram of randomization of patients in treatment groups, follow-up and analysis process. The study comprised 20 patients, 14 females (70 %) and 6 males (30 %), with 82 cutaneous lesions, 41 lesions in the *C. fistula* group and 41 lesions in the placebo group (Table 1). Nineteen out of 20 patients completed the trial and one patient left the study due to self-request. Table 2 displays characteristics of the patients and their lesions. The average diameter of erosions did not significantly vary between the two groups before treatment (7.37±3.97 vs 7.36±4.99 cm; p=0.57). The EI in the *C. fistula* fruit gel group was significantly higher than that of the placebo group both on day 10 (65±28vs 30±34; p*=*0.001) and at the end of the study (91±22 vs 69±49; p=0.003). As shown in Table 3, the average diameter of erosions after treatment was lower in the *C. fistula* fruit gel group compared to the placebo group (0.65±1.94 vs 2.17±3.40 cm; p=0.003). The treatment was not associated with any significant side effects, except for a bearable transient burning sensation in one patient following *C. fistula* fruit gel application for the first 2 days, which did not cause the patient to discontinue the treatment. 

**Table 1:** Basic demographic characteristics of participants included in the current trial

**Gender **	**Female**	**Male**
**No (%)**	14(70%)	6(30%)
**Age (year) ± SD**	59±18	60±22
**Disease duration (months) ± SD**	10.53±15.77	1±0

**Table 2:** Detailed characteristics of patients and their lesions

**ID**	**Gender**	**Age** **(years)**	**Systemic therapy**	**Location**	
				**Drug**	**Placebo**
**1**	M	36	P+A	Arm	Arm
				Forearm	Forearm
**2**	F	62	P+A	Scapula	Scapula
**3**	F	52	P+A	Thigh	Thigh
**4**	F	37	P+MM	Leg	Leg
				Thigh	Thigh
**5**	F	62	P	Buttock	Buttock
**6**	F	32	P+A	Axilla	Axilla
**7**	M	26	P+A+MM	Arm	Arm
				Axilla	Axilla
**8**	F	62	P+A	Axillary, breast	Axillary, breast
				Breast	Breast
**9**	F	81	P+MM	Buttock	Buttock
**10**	M	84	P+ Dapsone	Low back	Low back
				Low back	Low back
**11**	F	69	P+MM	Breast	Breast
				Upper abdomen	Upper abdomen
**12**	F	39	A+ Dapsone	Lower breast	Lower breast
				Lower breast	Lower breast
**13**	M	63	P+A	Arm	Arm
**14**	M	73	P	Arm	Arm
				Forearm	Forearm
**15**	F	79	P+A	Arm	Arm
**16**	F	30	P+A	Low back	Low back
**17**	F	84	P+MM	Thigh	Thigh
**18**	F	66	P+A	Neck	Neck
**19**	M	79	P+A	Forearm	Forearm
				Forearm	Forearm

P: prednisolone; A: azathioprine; MM: mycophenolate mofetil.

**Table 3:** Mean lesion size and Epithelization Index in the *Cassia fistula *fruit gel and placebo gel groups

**Outcomes**	***C. fistula*** ** fruit gel** **Mean± SD**	**Placebo gel** **Mean± SD**	**P value**
**Lesions diameter (cm)**			
**Day 0**	7.37±3.97	7.36±4.99	0.57
**Day 10**	2.52±2.58	5.29±5.23	0.006
**Day 20**	0.65±1.94	2.17±3.40	0.003
**Epithelization Index**			
**Day10**	65.84±28.48	30.29±34.32	0.001
**Day20**	91.74±22.07	69.63±49.76	0.003

## Discussion

This study showed that topical use of *C. fistula* fruit gel can reduce the healing time of skin lesions; also, the EI in the treatment group was significantly higher than that of the placebo group indicating the efficacy of topical *C. fistula* fruit gel in alleviating the skin lesions of PV.

Treatment of patients with PV commonly includes systemic administration of corticosteroids combined with an adjuvant immunosuppressive drug or anti-CD20 monoclonal antibody (rituximab) (Reguiai et al., 2012 ▶) and intravenous immunoglobulin (Ahmed, 2001 ▶). However, the side effects of these drugs are the most frequent causes of morbidity and mortality. Patients with PV are vulnerable to skin infections that could lead to severe sepsis and death. For this reason, local skin care plays an important role in the management of PV and favorable treatment of these patients includes the use of various supportive therapies which can decrease the healing time of lesions and as a result, reduce the need for steroid administration (Tabrizi et al., 2007 ▶; Iraji and Banan, 2010 ▶). In this context, some studies have investigated the effects of topical application of medications for reduction of the time required for wound healing in PV erosions. The efficacy of nicotinamide and pimecrolimus 1% as local treatments of PV, has been studied in two independent trials. Nicotinamide, as an anti-inflammatory drug used in autoimmune-inflammatory diseases could increase EI (Iraji and Banan, 2010 ▶). Pimecrolimus 1% which is a mast cell and T cell inhibitor was significantly more effective than placebo in improving EI in patients with PV (Iraji et al., 2010 ▶). The new topical medications including epidermal growth factor are probably beneficial to patients with PV lesions (Tabrizi et al., 2007 ▶). However, the present study has the advantage of considering a larger sample size of patients (19 vs 8 or 11) and total erosions (84 vs 60 or 62), compared to other identical trials (Iraji and Banan, 2010 ▶). 

Traditional Persian Medicine (TPM), (also known as Iranian Traditional Medicine) (Heydari et al., 2015a ▶) which is known as “hekmat” (Longo et al., 2013 ▶) is based on knowledge of medieval Persian physicians such as Rhazes (925 CE), Haly Abbas (982 CE) and Avicenna (1037 CE) (Heydari et al., 2015b ▶) who were familiar with different dermatologic disorders (Atarzadeh et al., 2016a ▶) including bullous pemphigoid lesions (Mortazavi et al., 2001 ▶). Various natural topical remedies could be found in TPM resources for wound healing (Jaladat et al., 2015 ▶). They have been used for many years by the Iranian physicians for management of various skin lesions in humans. The therapeutic approach of Persian medicine is related to its specific classification for ulcers and swelling to Hot and Cold ones (Atarzadeh et al., 2017b ▶).* C. fistula* L. has been widely used in Traditional Iranian Medicine as a medicinal plant. It has been used both orally and topically following preparation in an oily vehicle which is called* az- zomad* in TPM (**Avicenna, 1037**; Tunakabuni, 2007 ▶) for hot skin lesions. It was also used in the folk medicine of other Asian countries (Ayyanar and Ignacimuthu, 2009 ▶) for such purposes. Medicinal properties of the fruit are probably attributed to the presence of biologically active compounds such as anthraquinones (rhein), flavonoids, and triterpenoids (lupeol) (Lee et al., 2001 ▶; Thirumal et al., 2012 ▶). Lupeol can suppress the immune system (Amini et al., 2010 ▶; Siddique and Saleem, 2011 ▶) and pro-inflammatory cytokines such as TNFα (Saleem, 2009 ▶). It has been proven that topical application of lupeol decreases cell infiltration level toward murine inflamed tissues (Fernández et al., 2001 ▶). It also exhibited a significantly high wound- healing potential in mice (Saleem, 2009 ▶). It has been indicated that, as a natural anthraquinone derivative, rhein effectively decreases tissue edema and free-radical production in rats with inflammatory conditions (Tsang et al., 2013 ▶). Antioxidant activity of *C. fistula* fruit pulp powder was studied both *in vitro* and *in vivo*. High phenolic and flavonoid content of *C. fistula* L. probably contributes to its high antioxidant activity (Rajagopal et al., 2013 ▶). Recent findings imply high potency of rhein in suppressing the synthesis of a number of inflammatory factors including LT B4 and C4 in macrophages (Guo et al., 2002 ▶)*. *Also, antibacterial and antifungal activities of extracts of *C. fistula*, have been investigated (Bhalodia et al., 2012 ▶). Importantly,* C. fistula *L. has significantly low toxicity (Bahorun et al., 2004 ▶). 

In the current study, no significant side effects following topical application of *C. fistula* fruit gel, were observed except in one patient who only showed a mild transient burning sensation. These results suggest that *C. fistula* fruit gel can be considered a good and safe adjuvant therapy for treatment of coetaneous PV lesions. However, we believe that our findings should be evaluated in future clinical trials in larger population and compared with other routinely used topical ointments such as corticosteroids, antibacterial drugs, or tacrolimus to offer a safer and more effective low-cost, topical treatment option for patients with PV.

The limitation of this study is its small sample size chosen based on the inclusion criteria, and lack of data regarding patient's satisfaction with the drug vs. placebo. Yet another limitation was drug standardization, which is needed for extension and repetition of the work and is recommended to be done in future.

Topical application of *Cassia fistula* fruit gel can be considered an effective adjuvant therapy in treatment of PV.
